# USF2-Mediated APOE-Cholesterol Signaling Promotes CTC Clustering and Metastasis in Non-Small Cell Lung Cancer

**DOI:** 10.3390/ijms27177821

**Published:** 2026-08-31

**Authors:** Zheng Li, Meng Cui, Li Sun, Hao Qin, Xiaoyong Shen, Wen Gao

**Affiliations:** 1 Department of Thoracic Surgery, Huadong Hospital, Fudan University, Shanghai 200040, China; lizheng3698@163.com (Z.L.); 18796200179@163.com (H.Q.); 2 Department of Pharmacy, Huadong Hospital, Fudan University, Shanghai 200040, China; 25261030002@m.fudan.edu.cn; 3 Department of Radiation Oncology, Huadong Hospital, Fudan University, Shanghai 200040, China; 21111280011@m.fudan.edu.cn

**Keywords:** circulating tumor cell clusters, Apolipoprotein E, cholesterol metabolism, lung cancer metastasis, USF2 transcription factor

## Abstract

Circulating tumor cell (CTC) clustering is a key driver of metastatic progression in non-small cell lung cancer (NSCLC), but the underlying molecular mechanisms remain largely unknown. Here, we performed single-cell proteomic sequencing on CTC clusters and individual CTCs isolated from lung cancer patients, identifying 912 proteins in total, of which 246 were significantly upregulated in clusters versus single CTCs. Enrichment analyses revealed a prominent involvement of lipid and cholesterol metabolism pathways. Apolipoprotein E (APOE) emerged as a central hub, exhibiting marked upregulation in CTC clusters. Functional experiments showed that cholesterol supplementation promoted cluster formation in NSCLC cell lines and accelerated metastatic dissemination in vivo, whereas APOE depletion impaired clustering capacity. Mechanistically, we identified USF2 as a potential transcriptional regulator of APOE through direct binding to the promoter, as supported by Chromatin Immunoprecipitation (ChIP) and reporter assays. Clinical validation in a cohort of 75 lung cancer patients demonstrated that elevated APOE expression was strongly associated with advanced pathological grade, distant metastasis, and poor survival. Taken together, these findings establish a novel USF2–APOE–cholesterol axis that governs CTC clustering and metastatic progression, positioning APOE as both a prognostic biomarker and a potential therapeutic target for NSCLC metastasis.

## 1. Introduction

Lung cancer remains the leading cause of cancer-related mortality worldwide, accounting for approximately 1.8 million deaths annually and representing nearly 18% of all cancer fatalities [1]. The five-year survival rate for metastatic lung cancer remains dismally low at less than 5%, with metastatic progression being the primary determinant of patient survival [2]. The metastatic cascade involves multiple sequential steps, including local invasion, intravasation, survival in circulation, extravasation, and colonization at distant sites. Among these critical processes, the behavior of circulating tumor cells (CTCs) in the bloodstream has emerged as a pivotal factor influencing metastatic efficiency and clinical outcomes [3,4]. The vast majority of individual CTCs—estimated at over 99.9%—undergo rapid apoptosis within hours of entering circulation due to hemodynamic shear stress, immune surveillance by natural killer cells and macrophages, and loss of cell–matrix interactions leading to anoikis. However, a rare subset of CTCs can form multicellular clusters comprising 2–50 cells, which exhibit dramatically enhanced metastatic potential with colonization capacity up to 50-fold greater than single cells [5].

CTC clusters demonstrate remarkable survival advantages during circulation through multiple mechanisms. They exhibit increased resistance to anoikis through enhanced cell–cell adhesion mediated by E-cadherin and other adhesion molecules, which maintain survival signaling cascades typically lost in isolated cells [6]. Additionally, CTC clusters show superior immune evasion capabilities, as the outer cells in clusters can shield inner cells from immune recognition and cytotoxic attack [7]. Clinical studies across breast, prostate, lung, and colorectal cancers have consistently demonstrated that CTC cluster presence correlates with advanced disease stage, increased metastatic burden, and significantly worse patient outcomes. In non-small cell lung cancer specifically, elevated CTC cluster counts are independently associated with shortened progression-free survival (hazard ratio: 2.1–3.4) and overall survival, establishing their robust prognostic significance [8,9]. Despite accumulating clinical evidence supporting the critical role of CTC clusters in metastatic progression, the fundamental molecular mechanisms governing their formation and maintenance remain poorly understood.

Recent technological advances in single-cell genomics, proteomics, and metabolomics have begun to illuminate the molecular landscape underlying CTC biology, revealing unexpected complexity in their metabolic reprogramming [10,11]. Lipid metabolism has emerged as a particularly important regulatory pathway supporting various aspects of cancer progression, including membrane biogenesis for rapid proliferation, energy storage and production through fatty acid oxidation, and generation of bioactive lipid mediators that modulate signaling pathways [12,13]. Cholesterol metabolism, representing a fundamental component of cellular lipid homeostasis, plays increasingly recognized multifaceted roles in cancer biology that extend far beyond its structural membrane functions. Cancer cells frequently exhibit profound dysregulation of cholesterol biosynthesis, uptake, and efflux to support their aggressive phenotype, with cholesterol-rich membrane microdomains serving as platforms for oncogenic signaling pathway activation [14].

Apolipoprotein E (APOE), a 34-kDa multifunctional glycoprotein primarily known for its role in lipid transport and cholesterol homeostasis, has recently garnered significant attention in cancer research. APOE mediates cellular cholesterol uptake through interactions with low-density lipoprotein receptor family members and has been associated with cancer risk and progression across multiple malignancies [15]. However, its specific functional role in CTC biology and potential contribution to cluster formation have remained completely unexplored. The transcriptional regulation of APOE involves upstream stimulatory factor 2 (USF2), a basic helix–loop–helix transcription factor that regulates genes involved in lipid metabolism and cellular stress responses, though its role in cancer metastasis has not been investigated [16].

Given the critical importance of CTC clusters in determining metastatic outcomes and the emerging roles of cholesterol metabolism in supporting cancer cell survival and dissemination, we hypothesized that APOE-mediated cholesterol metabolism may represent a previously unrecognized mechanism contributing to CTC cluster formation and stability [17]. To test this hypothesis, we employed comprehensive single-cell proteomic profiling to systematically identify molecular differences between CTC clusters and individual CTCs isolated from lung cancer patients. Our findings reveal a novel USF2–APOE–cholesterol metabolic axis that actively promotes CTC clustering, enhances metastatic potential, and demonstrates significant clinical relevance as both a prognostic biomarker and potential therapeutic target for preventing lung cancer metastasis.

## 2. Results

### 2.1. Proteomic Sequencing of CTC Clusters and Individual CTCs

**Figure 1:**
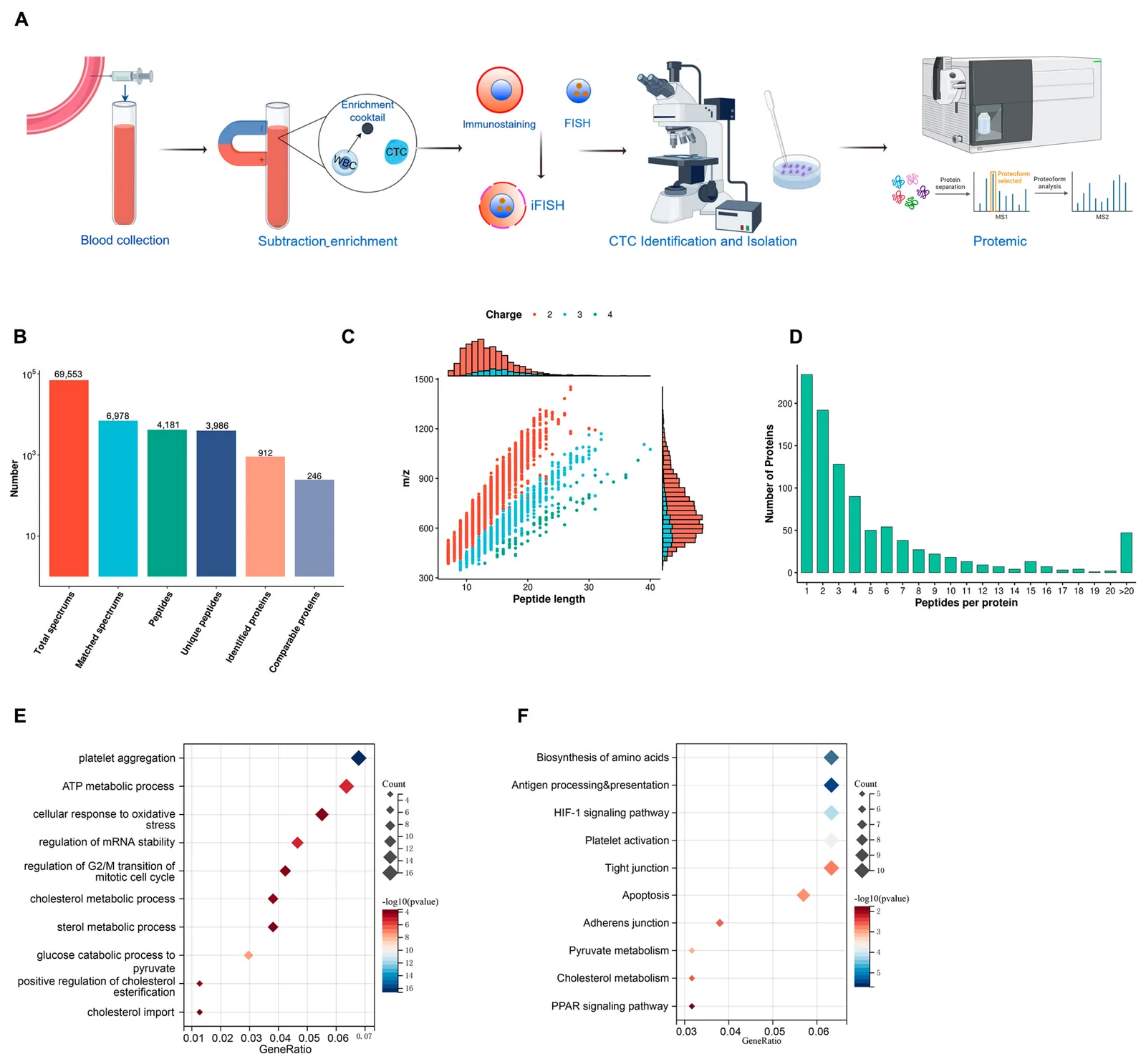
Proteomic analysis of individual CTCs and CTC clusters: (**A**) schematic representation of CTC proteomics; (**B**) overview of protein identification; (**C**) distribution of peptide lengths identified by mass spectrometry; (**D**) distribution of the number of peptides identified by mass spectrometry; (**E**) Gene Ontology (GO) enrichment analysis of paired proteins identified by mass spectrometry; (**F**) Kyoto Encyclopedia of Genes and Genomes (KEGG) enrichment analysis of the identified paired proteins.

**Figure A1:**
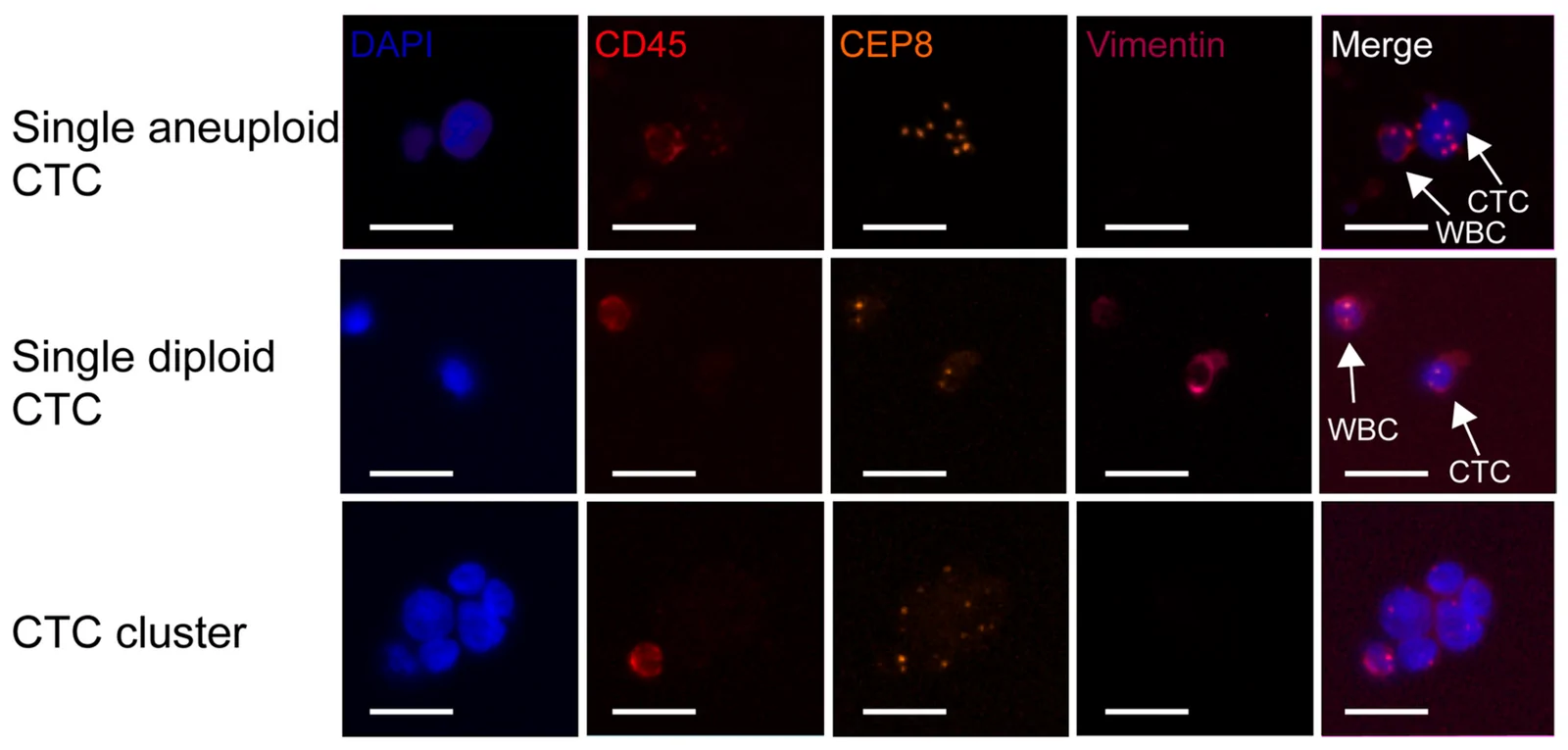
Immunofluorescence characterization of circulating tumor cells (CTCs) with different ploidy status. Fluorescent microscopy images showing three CTC types: single aneuploid CTC, single diploid CTC, and CTC cluster. Cells were stained with DAPI (blue) for nuclear visualization, anti-CD45 (red) for leukocyte identification, anti-CEP8 (orange) for chromosome enumeration, and anti-vimentin (magenta) for epithelial–mesenchymal transition marker expression. Scale bar = 50 μm.

To investigate the key mechanisms underlying the clustering of CTCs, we performed single-cell proteomic sequencing on CTC clusters and individual CTCs collected from the blood of lung cancer patients (Figure 1A). The CTC cluster identification is shown in Figure A1. A total of 11 CTC clusters (containing 45 CTCs) were microdissected from nine patient samples positive for CTC clusters. From the same samples, an equivalent number of 45 single CTCs were isolated as matched controls. Subsequent low-input proteomic profiling identified 912 proteins, of which 246 showed significant upregulation in CTC clusters relative to single CTCs (Figure 1B–D). Gene Ontology (GO) and Kyoto Encyclopedia of Genes and Genomes (KEGG) enrichment analyses of the identified 246 paired proteins revealed significant enrichment in lipid metabolism and cholesterol metabolism (Figure 1E,F). Additionally, Oil Red O staining of patient-derived CTC clusters and individual CTCs indicated a higher lipid content in the CTC clusters. These results suggest that lipid metabolism may play a crucial role in the development of CTC clusters from individual CTCs.

### 2.2. Cholesterol Metabolism Promotes CTC Clustering

**Figure 2:**
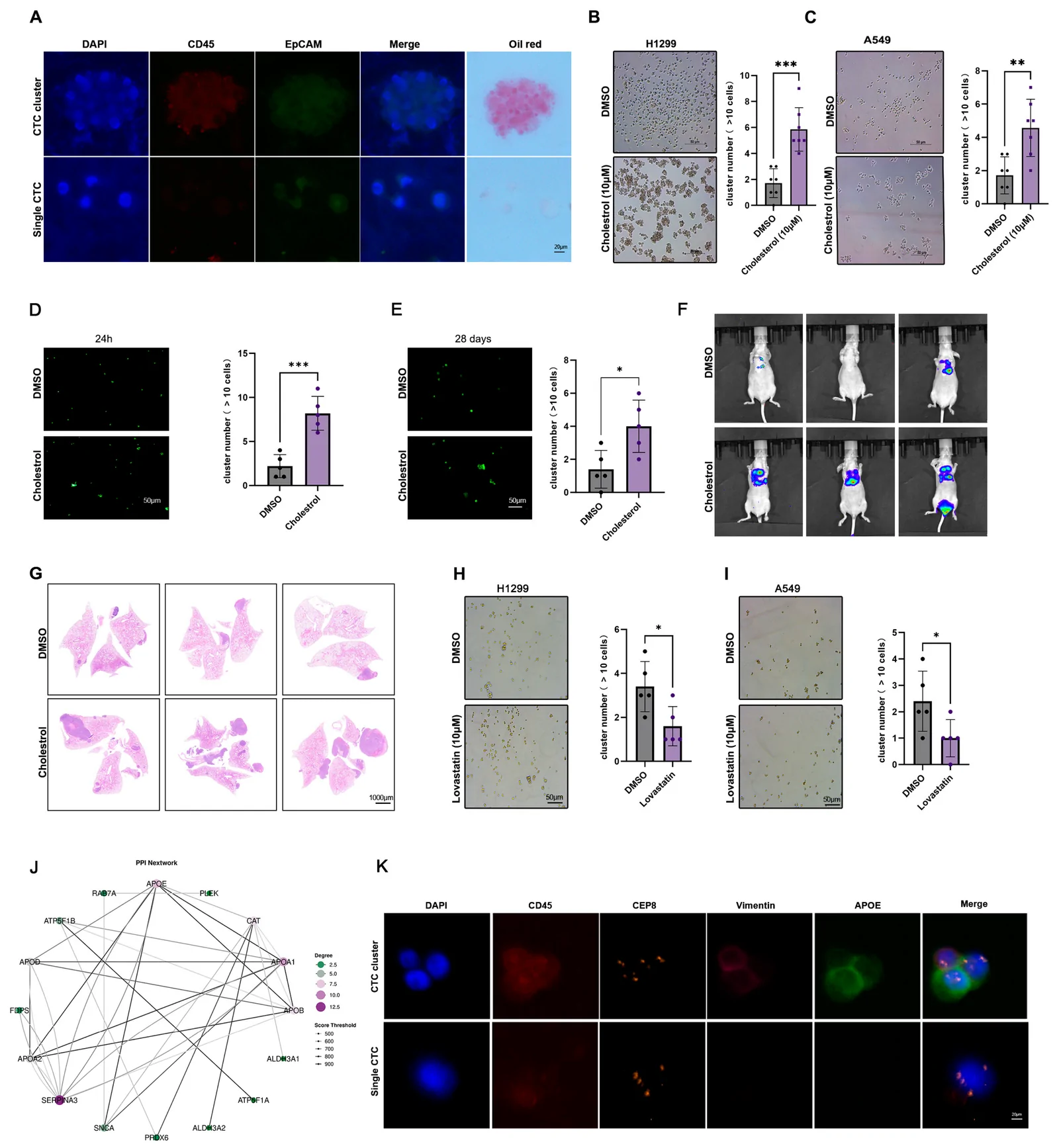
Cholesterol promotes the formation of CTC clusters: (**A**) representative images of Oil Red O staining analysis of individual CTCs and CTC clusters; (**B**,**C**) cluster formation of H1299 and A549 cells after 12 h of cholesterol treatment in ultra-low attachment culture plates; (**D**,**E**) representative images of circulating tumor cells (CTCs) and their clusters in the blood of mice at 24 h and 28 days of tumor modeling (cholesterol-treated or untreated); (**F**) in vivo imaging showing that cholesterol treatment promotes tumor formation in mice (*n* = 6 mice per group; representative images from 3 of 6 mice per group.); (**G**) representative hematoxylin and eosin (HE) staining of lung tumors in mice; (**H**,**I**) cluster formation of H1299 and A549 cells after 12 h of lovastatin treatment in ultra-low attachment culture plates; (**J**) protein–protein interaction (PPI) network diagram of lipid metabolism-related proteins identified by mass spectrometry; (**K**) representative images of immunofluorescence staining of APOE in individual CTCs and CTC clusters (* *p* < 0.05, ** *p* < 0.01, *** *p* < 0.001).

The proteomic results indicated that lipid metabolism could significantly influence the clustering of CTCs. To validate this, we performed Oil Red O staining on CTC clusters and individual CTCs, which confirmed a higher lipid content in the CTC clusters (Figure 2A). Cholesterol metabolism is one of the most prevalent lipid metabolic pathways. Therefore, we investigated the impact of cholesterol metabolism on CTC clustering. We added cholesterol (MedChemExpress, Shanghai, China, HY-N0322; 10 μM, 12 h) to the culture medium under suspension culture conditions, and the results demonstrated that cholesterol increased the number of clusters formed by non-small cell lung cancer (NSCLC) cell lines H1299 and A549 (Figure 2B,C). In a tail vein metastasis model, we collected blood samples from mice 24 h and 28 days after tail vein injection of A549 cells labeled with luciferase/GFP and quantitatively analyzed the number of circulating tumor cell (CTC) clusters and individual CTCs. The results showed that the number of CTC clusters in the cholesterol-treated group was significantly increased at both 24 h and 28 days (Figure 2D,E). In vivo imaging and lung tissue section analysis indicated that intraperitoneal injection of cholesterol enhanced systemic metastasis of A549 cells labeled with luciferase/GFP (Figure 2F,G). Further analysis using the cholesterol inhibitor inhibited the clustering ability of H1299 and A549 cells in vitro (Figure 2H,I). To further evaluate the role of cholesterol biosynthesis, we additionally performed cluster formation assays in the presence of the mevalonate pathway inhibitor lovastatin, which similarly suppressed cluster number in both lines (Figure 2H,I). These results are consistent with a contribution of cholesterol availability to CTC clustering, although we acknowledge that lovastatin may affect other mevalonate-dependent processes. These results suggest that cholesterol can promote the clustering of circulating tumor cells in the blood, thus potentially facilitating tumor metastasis and colonization, although additional studies are needed to further define the underlying mechanisms. To identify key genes involved in CTC cluster formation, we performed protein–protein interaction network analysis on lipid metabolism-related genes identified in proteomics studies. The results showed that APOE is a core component of this network (Figure 2J). Immunofluorescence staining analysis of CTC clusters and individual CTCs showed that APOE expression was significantly increased in CTC clusters (Figure 2K).

### 2.3. APOE-Mediated Cholesterol Metabolism Promotes CTC Clustering

**Figure 3:**
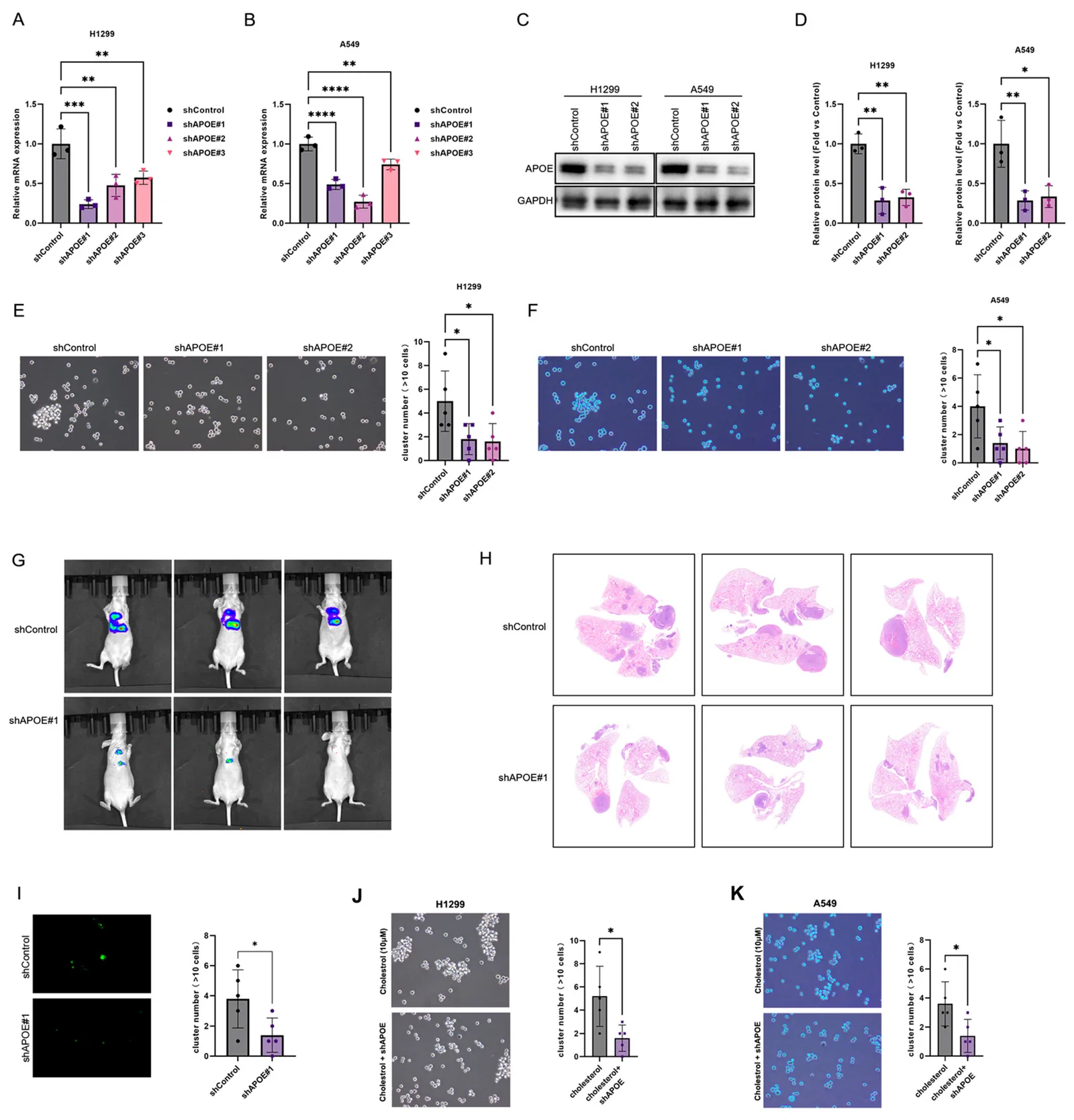
APOE knockdown inhibits CTC clustering and in vivo metastasis: (**A**–**D**) qPCR and Western blot analyses of APOE expression levels after lentiviral transfection; (**E**,**F**) cluster formation of H1299 and A549 cells in ultra-low attachment culture plates after 12 h of culture in control and APOE knockdown groups; (**G**) in vivo imaging showing lung tumor formation in APOE knockdown and control groups (*n* = 6 mice per group; representative images from 3 of 6 mice per group); (**H**) representative HE staining of lung tumors in mice; (**I**) representative images of individual CTCs and CTC clusters in the blood of mice treated or untreated with APOE knockdown; (**J**,**K**) cluster formation of H1299 and A549 cells in ultra-low attachment culture plates with or without cholesterol treatment (* *p* < 0.05, ** *p* < 0.01, *** *p* < 0.001, **** *p* < 0.0001).

**Figure A2:**
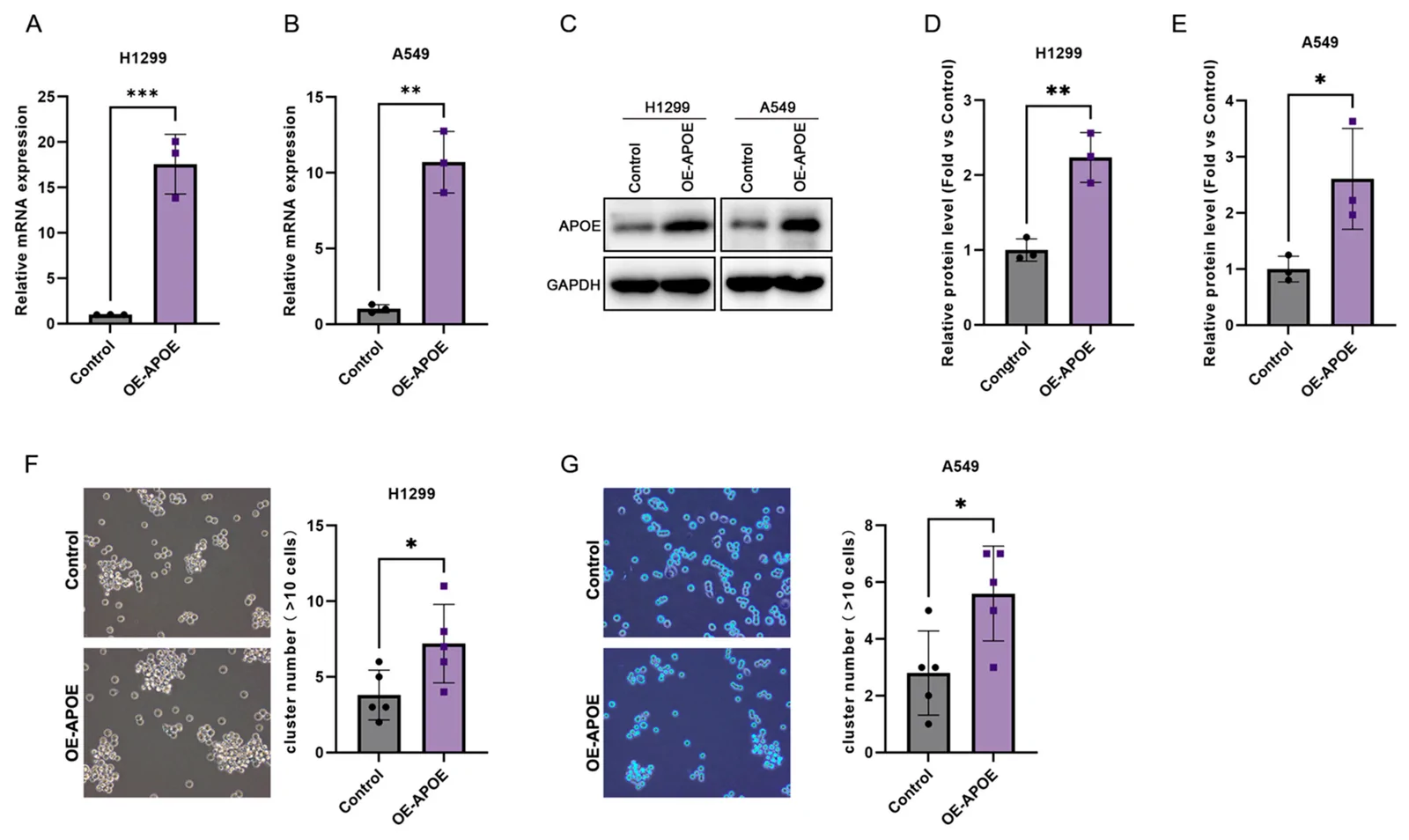
APOE overexpression promotes CTC clustering and in vivo metastasis: (**A**–**E**) qPCR and Western blot analyses of APOE expression levels following lentiviral transfection; (**F**,**G**) cluster formation of H1299 and A549 cells in ultra-low attachment culture plates after 12 h of culture in control and APOE overexpression groups (* *p* < 0.05, ** *p* < 0.01, *** *p* < 0.001).

We subsequently established stable cell lines with APOE knockdown and overexpression (Figure 3A–D). After culturing H1299 and A549 cells in ultra-low attachment plates for 12 h, we found that APOE knockdown significantly reduced the number of cell clusters (Figure 3E,F). Conversely, overexpression of APOE increased the number of clusters (Figure A2). In vivo experiments further demonstrated that APOE knockdown reduced the metastasis of tumor cells and the number of CTC clusters in the blood of mice (Figure 3G–I). Notably, APOE knockdown partially counteracted the clustering effect of cholesterol on the cells (Figure 3J,K). These results suggest that APOE is required for cholesterol-promoted CTC clustering and that its function may be linked to cholesterol-dependent mechanisms, although the precise molecular pathways remain to be fully elucidated.

### 2.4. USF2 Transcriptionally Activates APOE Expression

**Figure 4:**
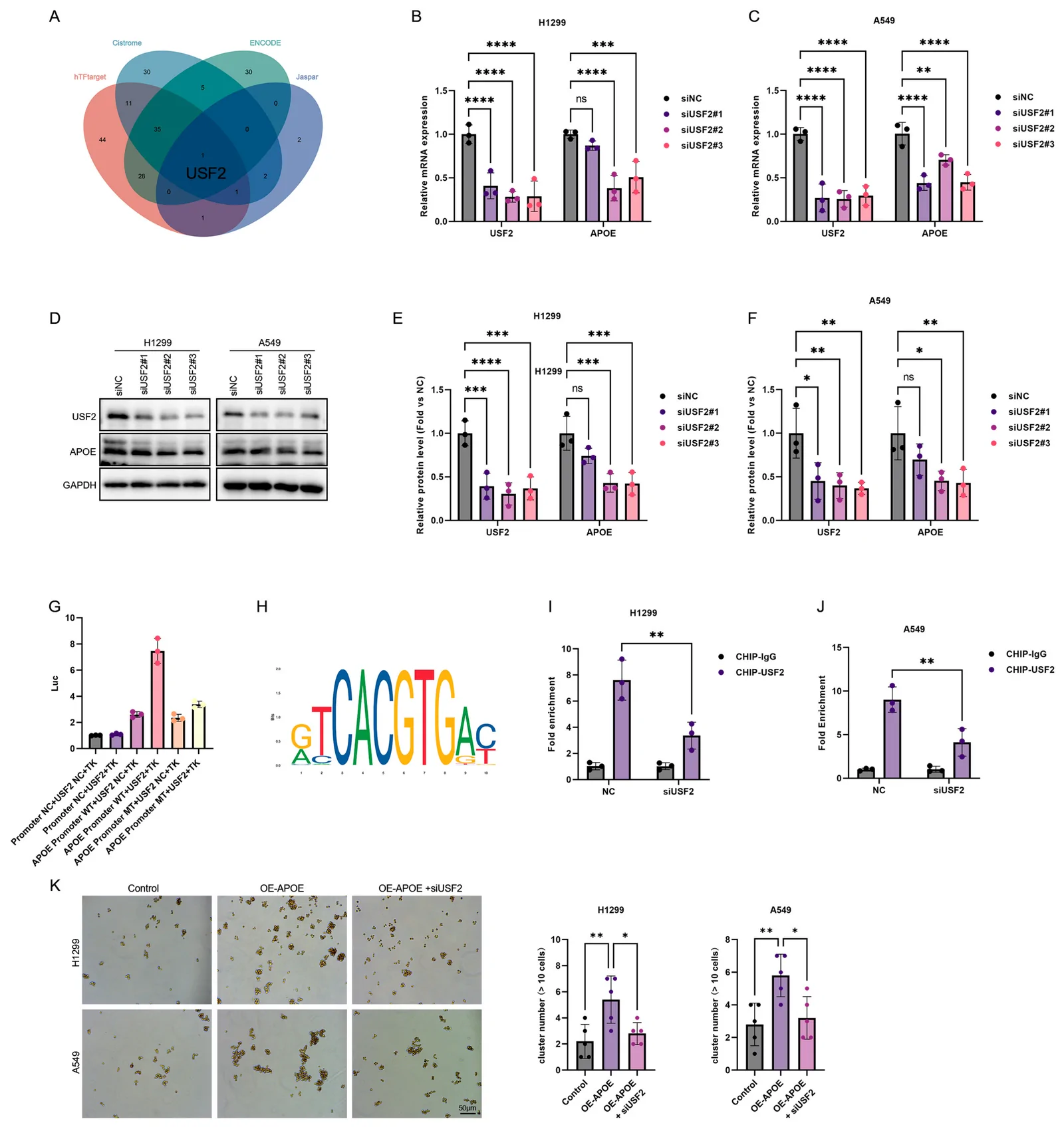
USF2 transcriptionally activates APOE expression: (**A**) Venn diagram of transcription factor databases; (**B**–**F**) qPCR and Western blot analyses of the effects of USF2 knockdown on APOE expression; (**G**) dual-luciferase reporter assay demonstrating the binding potential of USF2 to APOE; (**H**) binding sequences of the transcription factor; (**I**,**J**) ChIP-PCR results in H1299 and A549 cells following USF2 knockdown; (**K**) cluster formation of H1299 and A549 cells in ultra-low attachment culture plates with or without USF2 knockdown (ns *p* > 0.05, * *p* < 0.05, ** *p* < 0.01, *** *p* < 0.001, **** *p* < 0.0001).

Next, we sought to explore the upstream transcription factors regulating APOE expression. Analysis of multiple online databases revealed that USF2 may be a transcription factor that regulates APOE expression (Figure 4A). PCR and Western blot analyses showed that knockdown of USF2 resulted in a significant decrease in APOE expression at both protein and RNA levels (Figure 4B–F). Dual-luciferase reporter assays indicated a strong binding potential between USF2 and APOE. Moreover, ChIP-PCR results demonstrated that USF2 knockdown reduced the binding potential of APOE to USF2 (Figure 4G–J). Furthermore, USF knockdown also blocked tumor clustering caused by APOE overexpression. These findings are consistent with a model in which USF2 binds to the APOE promoter to activate its transcription, thereby contributing to CTC clustering.

### 2.5. High APOE Expression Is Associated with Poor Clinical Prognosis

**Figure 5:**
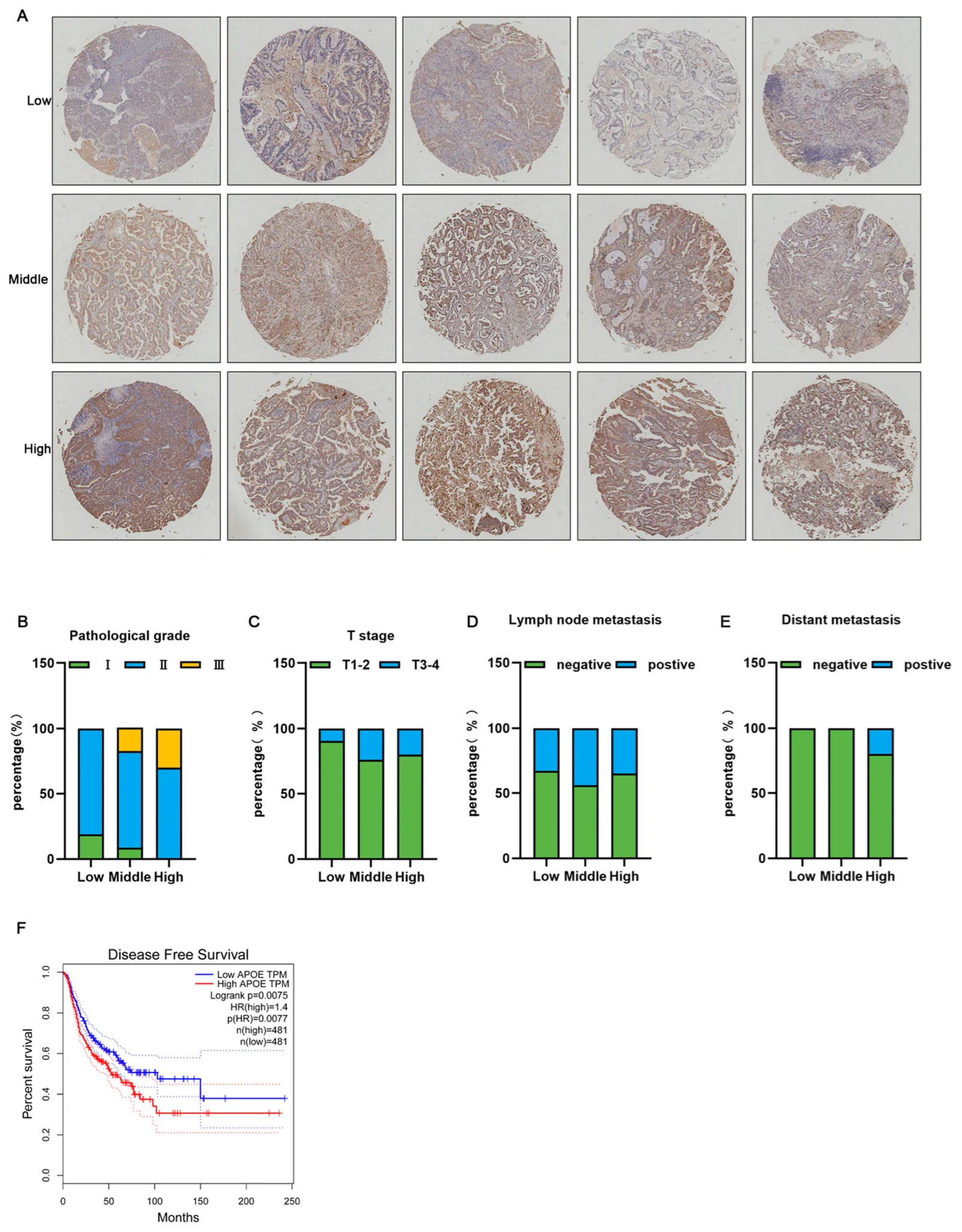
High APOE expression is associated with poor clinical prognosis: (**A**) representative images of APOE immunohistochemical staining in lung cancer tissues; (**B**–**E**) correlation statistics between APOE and clinical information; (**F**) survival curves of LUAD and LUSC from the TCGA database. For (**B**–**E**), patients were stratified by median H-score; for (**F**), patients were stratified by median APOE expression from TCGA datasets, the dotted line represents the 95% confidence interval.

To further investigate the clinical relevance of APOE, we analyzed a lung cancer tissue microarray consisting of 75 samples, and the patients’ information is listed in Supplementary Table S1. The results indicated that APOE expression was significantly correlated with pathological grade and distant metastasis. Patients with high APOE expression exhibited higher pathological grades and increased rates of distant metastasis (Figure 5A–E). Additionally, Kaplan–Meier analysis of the TCGA datasets revealed that NSCLC patients with elevated APOE levels had poor survival outcomes (Figure 5F). Taken together, our tissue microarray and TCGA-based analyses suggest that APOE expression levels are associated with tumor stage, lymph node metastasis, and poor survival in human NSCLC.

## 3. Discussion

The present study establishes a novel mechanistic framework for understanding CTC cluster formation through the identification of a USF2–APOE–cholesterol metabolic axis that critically regulates metastatic progression in lung cancer. Our comprehensive single-cell proteomic analysis revealed that CTC clusters exhibit distinct metabolic signatures characterized by enhanced lipid and cholesterol metabolism, with APOE emerging as a central regulatory hub. This finding aligns with accumulating evidence suggesting that metabolic reprogramming is fundamental to cancer cell survival and dissemination. Previous studies have demonstrated that CTCs must overcome multiple hostile conditions during their transit through the bloodstream, including nutrient deprivation, oxidative stress, and mechanical shear forces [18,19]. Our data suggest that APOE-mediated cholesterol metabolism provides a critical survival advantage by facilitating cluster formation, thereby enhancing resistance to anoikis and immune surveillance. The 50-fold increased colonization capacity observed in CTC clusters compared to individual CTCs underscores the clinical significance of this clustering mechanism and positions cholesterol metabolism as a previously underappreciated driver of metastatic efficiency.

The functional validation of cholesterol’s role in promoting CTC clustering represents a significant advance in our understanding of lipid metabolism in cancer metastasis [20]. Our demonstration that exogenous cholesterol supplementation enhanced cluster formation in vitro and accelerated metastatic dissemination in vivo provides compelling evidence for a direct causal relationship between cholesterol availability and metastatic potential. This observation is consistent with emerging literature implicating cholesterol metabolism in various aspects of cancer progression, including tumor growth, angiogenesis, and drug resistance [21,22]. Mechanistically, cholesterol-rich membrane microdomains, or lipid rafts, serve as platforms for oncogenic signaling pathway activation, including EGFR, PI3K/AKT, and Wnt signaling cascades that are crucial for cell survival and proliferation. Furthermore, cholesterol derivatives such as oxysterols function as bioactive signaling molecules that can modulate cellular processes, including migration and invasion [23]. Our findings extend these concepts to the specific context of CTC biology, suggesting that cholesterol metabolism not only supports individual cancer cell survival but also facilitates the formation of multicellular structures that dramatically enhance metastatic success.

The identification of APOE as the central mediator of cholesterol-induced CTC clustering provides important mechanistic insights into this process. APOE’s established role in lipid transport and cholesterol homeostasis positions it ideally to coordinate cellular cholesterol metabolism in response to environmental conditions [24]. Our demonstration that APOE knockdown significantly impaired clustering capacity while overexpression promoted cluster formation establishes a direct functional relationship between APOE expression levels and metastatic potential. We acknowledge that the APOE–cholesterol connection in this study is largely correlative. Direct evidence of APOE-mediated cholesterol uptake/efflux or LDLR involvement is not provided. Future studies are needed to dissect the precise pathways through which APOE coordinates cholesterol metabolism to promote CTC clustering. This finding is particularly noteworthy given that APOE polymorphisms have been associated with cancer risk and progression across multiple malignancies, including glioblastoma, breast cancer, and Alzheimer’s disease-related cancers [25,26,27]. However, our study is the first to demonstrate APOE’s specific role in CTC biology and metastatic dissemination. The partial rescue of cholesterol-induced clustering by APOE knockdown suggests that while APOE is a critical mediator, additional cholesterol-responsive pathways may also contribute to this process. This complexity highlights the multifaceted nature of lipid metabolism in cancer and suggests that therapeutic strategies targeting cholesterol metabolism may need to address multiple pathways simultaneously. Cholesterol likely promotes CTC cluster survival through additional mechanisms beyond the APOE axis. Cholesterol-rich lipid rafts in tumor cells facilitate EGFR, PI3K/AKT, and Wnt signaling, and oxysterols act as ligands of LXR and EBI2 to modulate migration and invasion. In the immune compartment, cholesterol polarizes tumor-associated macrophages toward an immunosuppressive APOE+ state and impairs NK-cell cytotoxicity, both of which may reduce immune clearance of CTC clusters. These parallel pathways are consistent with the partial rescue observed upon APOE knockdown and warrant further dissection.

The discovery that USF2 transcriptionally regulates APOE expression adds another layer of complexity to our understanding of this regulatory network. USF2 belongs to the basic helix–loop–helix family of transcription factors and is known to regulate genes involved in lipid metabolism, glucose homeostasis, and cellular stress responses [28,29]. Our chromatin immunoprecipitation and dual-luciferase reporter assays provide strong evidence for direct USF2-APOE transcriptional interaction, establishing USF2 as an upstream regulator of the cholesterol-clustering axis. This finding is significant because it identifies a potentially druggable transcriptional node that could be targeted to disrupt CTC clustering. Previous studies have shown that USF2 expression is dysregulated in various cancers and correlates with poor prognosis, consistent with our observations [30,31]. The USF2-APOE regulatory relationship also suggests that environmental or therapeutic interventions that modulate USF2 activity could indirectly influence cholesterol metabolism and metastatic potential, providing a rational basis for combination therapeutic approaches.

The clinical validation of our mechanistic findings through tissue microarray analysis and survival correlation studies demonstrates the translational relevance of the USF2–APOE–cholesterol axis. The strong associations between elevated APOE expression and advanced pathological grade, distant metastasis, and poor survival outcomes in 75 lung cancer patients provide compelling evidence for APOE’s prognostic value. These clinical correlations are consistent with previous studies in other cancer types that have implicated APOE in tumor progression and patient outcomes [32]. The association between elevated APOE expression and poor survival outcomes, as demonstrated by our Kaplan–Meier survival analyses of TCGA datasets, suggests that APOE could serve as a potential biomarker for risk stratification, although multivariable analysis in larger independent cohorts is needed to confirm its independent prognostic value. Our findings also suggest that APOE may represent a therapeutic target for preventing metastatic progression. Importantly, our findings have immediate clinical implications, as cholesterol-lowering therapies, including statins, are already FDA-approved and widely used for cardiovascular disease management. Recent epidemiological studies have suggested that statin use is associated with reduced cancer incidence and improved outcomes in several cancer types, potentially through cholesterol-dependent mechanisms [33,34]. Our mechanistic insights provide a rational framework for repurposing these agents as anti-metastatic therapies, particularly in patients with high APOE expression. We acknowledge that lovastatin inhibits the mevalonate pathway globally. Therefore, the observed effects should be interpreted as supporting, rather than proving, a cholesterol-specific mechanism. Nevertheless, our complementary approaches—including cholesterol supplementation, Oil Red O staining, and proteomic identification of APOE as a central hub in lipid metabolism—collectively support a role for cholesterol availability in CTC clustering. Future studies incorporating direct cholesterol measurements and rescue experiments will be required to further dissect the mechanism. The tail-vein injection model used in this study primarily reflects experimental colonization rather than the full spontaneous metastatic process.

The therapeutic potential of lipid-lowering agents in lung cancer treatment extends beyond traditional statin therapy. While statins are primarily recognized for their cardiovascular benefits through cholesterol reduction and plaque stabilization, emerging clinical evidence demonstrates significant anti-tumor properties. Statin therapy has been associated with reduced incidence of endometrial, ovarian, esophageal, gastric, and colorectal cancers, while also decreasing mortality rates in patients with esophageal, gastric, colorectal, prostate, and breast cancers [35,36]. As HMG-CoA reductase inhibitors, statins competitively inhibit the rate-limiting enzyme in the mevalonate pathway, thereby reducing both pathway activity and cholesterol biosynthesis. This disruption of cholesterol metabolism directly impacts lipid raft formation and membrane fluidity, which are crucial for tumor cell proliferation, migration, and signal transduction. Studies have shown that simvastatin inhibits tumor cell proliferation and migration by reducing lipid raft content through mevalonate pathway suppression [37]. Additionally, PCSK9 inhibitors such as evolocumab represent a novel therapeutic approach targeting cholesterol metabolism. PCSK9 normally promotes degradation of LDL receptors, and its inhibition enhances cholesterol uptake and clearance. A recent phase II clinical trial (ChiCTR2400086014) investigated the application of PCSK9 inhibitor recaticimab combined with immunotherapy in perioperative treatment of resectable NSCLC, providing important evidence for the translational potential of PCSK9-targeted therapy from cardiovascular to oncological applications [38]. Given our findings that APOE-mediated cholesterol metabolism drives CTC clustering, PCSK9 inhibitors may offer complementary anti-metastatic effects by modulating cholesterol homeostasis through a distinct mechanism from statins. The combination of our mechanistic insights with existing clinical evidence for lipid-lowering agents suggests promising opportunities for repurposing these therapies in lung cancer management, particularly for preventing metastatic progression in high-risk patients. We acknowledge that our clinical validation is limited by the small cohort size (*n* = 75) and the small number of M-positive cases (*n* = 4). Multivariable analysis was not feasible. Therefore, the prognostic value of APOE observed here should be considered preliminary, and larger cohorts are required for validation. Future clinical trials evaluating the efficacy of cholesterol-targeted interventions in preventing CTC clustering and metastatic progression are warranted based on our preclinical findings.

## 4. Materials and Methods

### 4.1. Cell Lines

The non-small cell lung cancer cell lines A549 and H1299 were purchased from Wuhan Puno Biotechnology Co., Ltd. (Wuhan, China). H1299 cells were cultured in 10% fetal bovine serum (FBS) and 1% penicillin–streptomycin in 1640 medium, while A549 cells were cultured in 10% FBS and 1% penicillin–streptomycin in DMEM. Cell cultures were maintained in a humidified incubator at 5% CO_2_.

### 4.2. Cholesterol and Lovastatin Treatment

Cholesterol (MedChemExpress, HY-N0322, Shanghai, China) was dissolved in anhydrous ethanol as a stock solution and diluted with culture medium to a final working concentration of 10 μM. Cells were treated with cholesterol for 12 h under suspension culture conditions. For lovastatin treatment, cells were treated with lovastatin at 10 μM for 12 h. Control cells received an equal volume of vehicle.

### 4.3. Mice and In Vivo Metastasis Assay

Six- to eight-week-old female BALB/c nude mice were purchased from Jiangsu Jicui Yikang Biotechnology Co., Ltd. (Nanjing, China) and housed in SPF-grade facilities at Fudan University. For each experimental group, 6 mice were used. For tail-vein injection, 2 × 10^6^ A549-Luc/GFP cells in 100 μL PBS were injected via the lateral tail vein. Cholesterol (MCE, HY-N0322) was dissolved in anhydrous ethanol as a stock solution and diluted with sterile saline to a final concentration containing <1% ethanol, then administered intraperitoneally at 5 mg/kg every 2 days for 28 days. The control group received an equal volume of vehicle (saline containing the same final concentration of ethanol). Mice were randomly assigned to groups, and investigators were blinded during outcome assessment. Humane endpoints were predefined, and no animals were excluded. All procedures were approved by the Animal Care and Use Committee of Fudan University (protocol code A20260066).

### 4.4. Cell Transfection

Lentiviral constructs for APOE were generated by Shanghai Jikai Gene Biotechnology Co., Ltd. (Shanghai, China). Cells were plated at 30% density in six-well plates one day prior to transfection. The next day, lentivirus and transfection reagents were added. After 12 h, the medium was replaced with fresh culture medium. Seventy-two hours post-transfection, the medium was supplemented with 4 μg/mL puromycin for one week to select stable cell lines.

### 4.5. Circulating Tumor Cell Enrichment

Circulating tumor cells (CTCs) were isolated from whole blood using subtraction enrichment methodology according to the manufacturer’s protocol (Cytelligen, San Diego, CA, USA) [39]. Venous blood samples (6 mL per patient) were collected from the antecubital vein into acid–citrate–dextrose (ACD) anticoagulant tubes prior to treatment initiation. Blood samples underwent initial centrifugation at 200× *g* for 15 min at room temperature (RT). The supernatant was removed, and pelleted cells were resuspended in 3 mL of CTC buffer before being layered onto a non-hematopoietic cell separation matrix in 50 mL conical tubes. Samples were subsequently centrifuged at 350× *g* for 6 min at RT. The buffy coat layer was carefully collected and transferred to a fresh 50 mL tube. For leukocyte depletion, the cell suspension was incubated with 300 μL of anti-CD45 monoclonal antibody-conjugated magnetic beads for 20 min at RT with gentle mixing. Magnetic separation was performed using a commercial magnetic separation stand (Promega, Madison, WI, USA) to remove leukocyte-bound magnetic beads. The unbound cell fraction was collected, centrifuged, and concentrated to approximately 100 μL, yielding a CTC-enriched suspension with depleted white blood cells for subsequent downstream analysis.

### 4.6. Immunostaining–Fluorescence In Situ Hybridization (i-FISH)

Immunofluorescence FISH (i-FISH) analysis was performed according to the manufacturer’s protocol (Cytelligen, San Diego, CA, USA) [40]. CTC-enriched cell suspensions obtained through subtractive white blood cell depletion were treated with 2 μL citrate buffer, gently mixed, and incubated for 10 min at room temperature. A dual-staining cocktail was prepared by combining 1 μL anti-CD45 antibody and 1 μL anti-vimentin antibody in 200 μL blood cell analysis diluent. For validation of APOE expression in CTC clusters, the staining cocktail was supplemented with 1 μL anti-APOE antibody (catalog number CL488-66830; Proteintech, Rosemont, IL, USA). The antibody cocktail was vortex-mixed with the cell suspension and incubated for 20 min at room temperature in the dark. Following incubation, cells were washed with 5 mL cell rinse buffer and resuspended in 100 μL. Cell fixation was performed by adding 100 μL of Cytelligen Fixative to the suspension. Fixed cells were applied to manufacturer-specific slides, dried overnight at 30 °C, and prepared for fluorescence in situ hybridization. For chromosomal analysis, 10 μL of Spectrum Green-conjugated chromosome 8 centromeric probe (CEP8; Vysis, Abbott Laboratories, Abbott Park, IL, USA) was applied to the prepared slides. DNA denaturation was performed at 76 °C for 10 min, followed by probe hybridization at 37 °C for 3 h under coverslips in a humidified chamber. Post-hybridization washing was conducted in 2 × saline–sodium citrate (SSC) buffer containing 0.3% NP-40 at 72 °C to remove unbound probe. Slides were air-dried and counterstained with 10 μL DAPI (4′,6-diamidino-2-phenylindole) in antifade mounting medium before coverslip application. Slides were stored at 4 °C in the dark until microscopic analysis.

### 4.7. Scanning and Identification of CTCs and CTC Clusters

Cytelligen-formatted slides were scanned using the Metafer-iFISH^®^ automated imaging platform (Carl Zeiss, Oberkochen, Germany). Automated classification was performed based on predefined criteria: CTCs were defined as DAPI+/CD45-/Vimentin+/- cells with aneuploid Chr8 or DAPI+/CD45-/Vimentin+ cells with diploid Chr8; CTC clusters as ≥2 aggregated CTCs; and WBCs as DAPI+/CD45+ cells with diploid Chr8 (Figure 2H). All samples were independently reviewed by two experienced researchers. For in vitro clustering assays and mouse blood samples, clusters were defined as aggregates containing > 10 cells, based on a previous study [41].

### 4.8. Low-Input Cell Proteomic Analysis

#### 4.8.1. Isolation of Single CTCs and CTC Clusters

Processed specimens were mounted on a single-cell picking system (NMSCM) with CellPick Buffer-2 applied to the sample area. Target CTCs and CTC clusters were relocated using coordinate mapping from pre-acquired scan data. Surrounding WBCs were removed using glass capillaries, followed by gentle detachment and aspiration of target cells (0.5–1 μL volume). Cellular contents were dispensed into PCR tubes containing 3 μL PBS. Single CTCs and CTC clusters were collected into separate tubes with equivalent cell numbers maintained for comparative proteomic analysis.

#### 4.8.2. Trypsin Digestion

Samples were resuspended in lysis buffer, sonicated using a contactless high-intensity ultrasonic processor (Scientz, Ningbo, China) for 3 min, and heated at 95 °C for 10 min. Trypsin (Promega) was added for overnight protein digestion at 37 °C. Peptides were reduced with dithiothreitol (DTT, Sigma, St. Louis, MO, USA) at 56 °C for 30 min, followed by alkylation with iodoacetamide (IAA, Sigma) for 15 min in darkness. Finally, peptides were desalted using C18 ZipTips (Millipore, Burlington, MA, USA) according to the manufacturer’s instructions.

#### 4.8.3. LC-MS/MS Analysis

Following dissolution in mobile phase A (0.1% formic acid, 2% acetonitrile/water), tryptic peptides were loaded onto a custom-packed reversed-phase analytical column (25 cm × 100 μm i.d.). Separation was performed on a NanoElute UHPLC system (Bruker Daltonics) using a binary gradient at 100 nL/min: initially 7% B (0.1% formic acid/acetonitrile), increased to 16% B over 4 min, 16–26% B from 4 to 12 min, 26–40% B from 12 to 15 min, 40–80% B from 15 to 17 min, and maintained at 80% B from 17 to 20 min. Eluted peptides underwent electrospray ionization at 1.75 kV prior to timsTOF Pro MS analysis (Bruker, Bremen, Germany). MS/MS spectra (100–1700 m/z) were acquired in PASEF mode with precursor charge states 0–5 selected for fragmentation, collecting 3 PASEF-MS/MS scans per cycle under 30 s dynamic exclusion. Protein relative quantification was performed using iBAQ intensity values, and the resulting relative quantitative values were median-normalized across samples to correct for systematic loading differences.

#### 4.8.4. Database Search

MS/MS spectra were analyzed using MaxQuant (v1.6.15.0) with search parameters configured as follows: Spectra were queried against the Homo_sapiens_9606_SP_20220107.fasta database (20,376 entries) supplemented with reverse decoy sequences and common contaminants. Trypsin/P digestion allowing ≤2 missed cleavages was specified, with a minimum peptide length set at 7 residues and a maximum of 5 modifications per peptide. Precursor mass tolerances were 20 ppm in both initial and main searches, with fragment ion tolerance at 20 ppm. Carbamidomethylation of cysteine was designated as a fixed modification, while protein N-terminal acetylation and methionine oxidation were variable modifications. False discovery rates (FDR) were controlled at <1% for protein, peptide, and PSM identifications.

#### 4.8.5. Quantitative and Statistical Analysis for Proteomics

Differentially expressed proteins between CTC clusters and individual CTCs were identified by calculating fold changes (FCs) based on the median-normalized relative quantitative values. Proteins with a fold change ≥ 1.5 were considered significantly upregulated in CTC clusters, and those with a fold change ≤ 1/1.5 (i.e., <0.67) were considered significantly downregulated. For functional enrichment analysis (Gene Ontology and KEGG pathways), Fisher’s exact test was used to assess the significance of enrichment. To correct for multiple hypothesis testing, the Benjamini–Hochberg false discovery rate (FDR) method was applied, and an adjusted *p* value < 0.05 was considered statistically significant. For missing quantitative values, proteins with valid measurements in fewer than 70% of samples were excluded from subsequent analysis. All proteomic data processing and statistical analyses were performed using R software (version 3.5.2).

### 4.9. Oil Red O Staining

Single CTCs and CTC clusters were washed once with PBS. Subsequently, 4% paraformaldehyde fixation solution (P0099) or 10% formaldehyde solution was added for 10 min, followed by two washes with PBS. An appropriate volume of Oil Red O staining solution was applied to cover the cells for 10–20 min. After staining, the cells were observed under a microscope. CTCs were identified using immunofluorescence staining with a cocktail containing CD45 (1 μL) and EpCAM (1 μL) antibodies in 200 μL blood cell analysis diluent. Staining procedures followed the protocol described in the CTC Immunofluorescence Identification section, excluding FISH processing. CTCs were defined as DAPI^+^/EpCAM^+^/CD45^−^ cells.

### 4.10. Clustering Assay

To assess clustering, cultured cells were digested into single-cell suspensions and seeded at a density of 1 × 10^4^ cells/mL in ultra-low attachment 24-well plates (500 μL per well). For cholesterol treatment, cells were incubated with cholesterol (MedChemExpress, HY-N0322, Shanghai, China) at a final concentration of 10 μM for 12 h under suspension culture conditions. After 12 h of incubation, clustering was quantified by imaging 5 randomly selected, non-overlapping fields per well (selected along the horizontal and vertical axes). The number of large clusters, defined as aggregates containing > 10 cells, was counted per field. Each experiment included three technical replicates (wells) and was independently repeated three times (biological replicates).

### 4.11. Quantitative Reverse Transcription Polymerase Chain Reaction (qRT-PCR) Analysis

Total RNA was isolated using TRIzol reagent (Invitrogen, Carlsbad, CA, USA) according to the manufacturer’s protocol and reverse transcribed into complementary DNA using the PrimeScript RT Reagent Kit (TaKaRa, Kusatsu, Shiga, Japan). qRT-PCR was performed using a TaKaRa Step One Plus Real-Time PCR system (Applied Biosystems, Foster City, CA, USA) with SYBR Premix Ex Taq (TaKaRa). Gene expression was calculated using the 2^−ΔΔCT^ method, with GAPDH serving as the endogenous control for target gene expression.

### 4.12. Western Blot

H1299 and A549 cells were washed twice with phosphate-buffered saline (PBS) prior to harvesting. Cell lysis was performed using a commercial lysis buffer (Beyotime, Shanghai, China) with continuous agitation at 4 °C for 30 min. After lysis, cellular debris was pelleted via high-speed centrifugation (12,000× *g* for 15 min), and the supernatant containing soluble proteins was collected. Protein concentrations were quantified using a bicinchoninic acid (BCA) assay kit (Thermo Fisher Scientific, Waltham, MA, USA) following the manufacturer’s instructions. For immunoblotting, 25 μg of total protein per sample was denatured in Laemmli buffer at 95 °C for 5 min and resolved by sodium dodecyl sulfate–polyacrylamide gel electrophoresis (SDS-PAGE). Proteins were then transferred electrophoretically onto polyvinylidene fluoride (PVDF) membranes (Millipore) using a semi-dry transfer system. Membranes were blocked with 5% non-fat milk in Tris-buffered saline containing 0.1% Tween-20 (TBST) for 1 h at room temperature, followed by overnight incubation at 4 °C with primary antibodies targeting cIAP2. After three washes with TBST, membranes were incubated with horseradish peroxidase (HRP)-conjugated secondary antibodies for 1 h at RT. Protein bands were visualized using an enhanced chemiluminescence (ECL) detection system (Beyotime), and images were captured using a gel documentation system.

### 4.13. Chromatin Immunoprecipitation (ChIP) PCR

ChIP assays were performed using a commercial kit (Beyotime, China). Cells were crosslinked with 1% formaldehyde, and chromatin was sheared by sonication to 200–500 bp fragments. Chromatin was immunoprecipitated overnight with anti-USF2 antibody (Santa Cruz, sc-862, Dallas, TX, USA) or normal rabbit IgG (negative control). After reversal of crosslinks, purified DNA was analyzed by qPCR using primers flanking the predicted USF2-binding sites. Primer sequences were as follows: forward, 5′-CCTGGGCTGGGATGGAA-3′; reverse, 5′-GTCGCCTGTCACTGGGAA-3′. Enrichment was calculated relative to input chromatin and normalized to IgG control.

### 4.14. Dual-Luciferase Reporter Assay

The human APOE promoter region (−1500 to +100 bp) containing the putative USF2-binding site was cloned into the pGL3-basic luciferase reporter vector (Promega). A mutant construct with the USF2-binding motif deleted served as the control. H1299 and A549 cells were co-transfected with the wild-type or mutant reporter plasmid, USF2 expression plasmid (or empty vector), and pRL-TK Renilla internal control. Firefly and Renilla luciferase activities were measured 48 h post-transfection. Relative luciferase activity was calculated as the ratio of firefly to Renilla and normalized to the empty vector control.

### 4.15. Tissue Microarray and Immunohistochemistry

A commercial TMA containing 75 lung cancer cases with matched adjacent tissues (150 cores) was purchased from Shanghai Outdo Biotech Co., Ltd., Shanghai, China. TMA sections were incubated with anti-APOE antibody (Proteintech, 66830-1-Ig, 1:200, Rosemont, IL, USA) and visualized by DAB. APOE expression was scored independently by two blinded pathologists using an H-score (intensity 0–3 × percentage of positive cells 0–100%), and cases were stratified into high- and low-expression groups by the median H-score.

### 4.16. TCGA Database Analysis

To evaluate the prognostic value of APOE expression, Kaplan-Meier survival analysis was performed using RNA-seq data and corresponding clinical information from The Cancer Genome Atlas (TCGA) LUAD and LUSC cohorts. Patients were stratified into high- and low-expression groups based on the median APOE expression level. Survival curves were generated using the Kaplan–Meier method and compared by the log-rank test.

### 4.17. Statistical Analysis

Data analysis was performed using GraphPad Prism 5.02 software (GraphPad Software, San Diego, CA, USA). Results are presented as mean ± SD. All in vitro experiments were independently repeated at least three times (biological replicates), with the well used as the statistical unit for clustering assays and the individual mouse for in vivo experiments. Comparisons between two groups were performed using an unpaired two-tailed Student’s *t*-test, and multiple group comparisons were performed using one-way ANOVA with Tukey’s post hoc test. Clinicopathological correlations were analyzed by Fisher’s exact test or chi-squared test. Kaplan–Meier curves were compared by the log-rank test. A *p* < 0.05 was considered statistically significant (*p* < 0.05, * *p* < 0.01, ** *p* < 0.001, **** *p* < 0.0001).

## 5. Conclusions

In summary, our study reveals a previously unrecognized USF2–APOE–cholesterol metabolic axis that critically governs circulating tumor cell clustering and metastatic progression in lung cancer. Through comprehensive single-cell proteomic profiling, we identified cholesterol metabolism as a key regulatory pathway distinguishing CTC clusters from individual CTCs, with APOE serving as the central mediator of this process. Functional validation demonstrated that cholesterol supplementation promotes CTC clustering and enhances metastatic dissemination, while APOE depletion significantly impairs these processes. The discovery of USF2 as the upstream transcriptional regulator of APOE provides mechanistic insights into this regulatory network and identifies potential therapeutic intervention points. Importantly, clinical validation in lung cancer patients establishes strong associations between elevated APOE expression and adverse clinical outcomes, including advanced pathological grade, distant metastasis, and poor survival. These findings position APOE as both a prognostic biomarker for risk stratification and a promising therapeutic target for anti-metastatic interventions. The identification of this cholesterol-dependent clustering mechanism offers new opportunities for repurposing existing cholesterol-lowering therapies as adjuvant treatments to prevent metastatic progression, potentially improving outcomes for lung cancer patients with high metastatic risk. We acknowledge that our clinical validation is limited by the small cohort size (*n* = 75) and the small number of M-positive cases (*n* = 4). Multivariable analysis was not feasible. Therefore, the prognostic value of APOE observed here should be considered preliminary, and larger cohorts are required for validation.

## Data Availability

The data that support the findings of this study are available from the corresponding author (Wen Gao) upon reasonable request: gao_wen@fudan.edu.cn; gaowen_chest@163.com.

## Supplementary Materials

The following supporting information can be downloaded at https://www.mdpi.com/article/10.3390/ijms27177821/s1.

## Abbreviations

The following abbreviations are used in this manuscript:

CTC	Circulating tumor cell
NSCLC	Non-small cell lung cancer
APOE	Apolipoprotein E
USF2	Upstream stimulatory factor 2
i-FISH	Immunofluorescence FISH
KEGG	Kyoto Encyclopedia of Genes and Genomes
