# Supplementary material for: USF2-Mediated APOE-Cholesterol Signaling Promotes CTC Clustering and Metastasis in Non-Small Cell Lung Cancer
Source: Int J Mol Sci. 2026 Aug 31;27(17):7821. doi: 10.3390/ijms27177821 (PMC13566695; doi:10.3390/ijms27177821)
Supplement: Supplementary file 1 [file ijms-27-07821-s001.zip › ijms-4365337-supplementary.pdf]

USF2-Mediated APOE-Cholesterol Signaling Promotes CTC Clustering and Metastasis in Non-Small Cell Lung Cancer

Table S1. Correlation of APOE expression with pathological grade, distant metastasis, and other clinical features in NSCLC patients (n = 75).

Variable	Overall, N = 75[1]	high, N = 20[1]	low, N = 21[1]	middle, N = 34[1]	p-value[2]
Pathological grade					0.022
I	7 (9.3%)	0 (0%)	4 (19%)	3 (8.8%)	
II	56 (75%)	14 (70%)	17 (81%)	25 (74%)	
III	12 (16%)	6 (30%)	0 (0%)	6 (18%)	
Gender					0.14
female	35 (47%)	8 (40%)	7 (33%)	20 (59%)	
male	40 (53%)	12 (60%)	14 (67%)	14 (41%)	
Age	63 (54, 68)	64 (56, 68)	63 (59, 72)	60 (52, 65)	0.4
T					0.5
T1-2	61 (81%)	16 (80%)	19 (90%)	26 (76%)	
T3-4	14 (19%)	4 (20%)	2 (9.5%)	8 (24%)	
N					0.7
negative	46 (61%)	13 (65%)	14 (67%)	19 (56%)	
postive	29 (39%)	7 (35%)	7 (33%)	15 (44%)	
M					0.004
negative	71 (95%)	16 (80%)	21 (100%)	34 (100%)	
postive	4 (5.3%)	4 (20%)	0 (0%)	0 (0%)	
Grade					0.2
grade 1	37 (49%)	8 (40%)	12 (57%)	17 (50%)	
grade 2	18 (24%)	5 (25%)	6 (29%)	7 (21%)	
grade 3	15 (20%)	3 (15%)	3 (14%)	9 (26%)	
grade 4	5 (6.7%)	4 (20%)	0 (0%)	1 (2.9%)	

[1]n (%); Median (IQR)

[2]Fisher's exact test; Pearson's Chi-squared test; Kruskal-Wallis rank sum test

Cases were stratified into high, middle, and low expression groups based on the median H-score.
